# Short and medium-term effects of the COVID-19 lockdowns on child and parent accelerometer-measured physical activity and sedentary time: a natural experiment

**DOI:** 10.1186/s12966-023-01441-1

**Published:** 2023-04-27

**Authors:** Russell Jago, Ruth Salway, Danielle House, Robert Walker, Lydia Emm-Collison, Kate Sansum, Katie Breheny, Tom Reid, Sarah Churchward, Joanna G. Williams, Charlie Foster, William Hollingworth, Frank de Vocht

**Affiliations:** 1 Centre for Exercise, Nutrition and Health Sciences, School for Policy Studies, University of Bristol, 8 Priory Rd, Bristol, BS8 1TZ UK; 2 Population Health Sciences, Bristol Medical School, University of Bristol, Bristol, BS8 2PS UK; 3 The National Institute for Health Research, Applied Research Collaboration West (NIHR ARC West), University Hospitals Bristol and Weston NHS Foundation Trust, Bristol, BS1 2NT UK; 4 NIHR Bristol Biomedical Research Centre, University Hospitals Bristol and Weston NHS Foundation Trust and University of Bristol, Bristol, UK; 5 Independent Public Member of the Project Team, Bristol, UK; 6 Communities and Public Health, Bristol City Council, Bristol, BS1 9NE UK

**Keywords:** Physical activity, Natural experiment, Children, COVID-19, Parents

## Abstract

**Background:**

The COVID-19 pandemic has resulted in marked impacts on children’s physical activity, with large reductions in moderate-to-vigorous physical activity (MVPA) reported during lockdowns. Previous evidence showed children’s activity levels were lower and sedentary time higher immediately post-COVID lockdown, while there was little change in parental physical activity. We need to know if these patterns persist.

**Methods:**

Active-6 is a natural experiment using repeated cross-sectional data conducted in two waves. Accelerometer data were collected on 393 children aged 10–11 and their parents from 23 schools in Wave 1 (June 2021-December 2021), and 436 children and parents from 27 schools in Wave 2 (January 2022-July 2022). These were compared to a pre-COVID-19 comparator group (March 2017-May 2018) of 1,296 children and parents in the same schools. Mean minutes of accelerometer-measured MVPA and sedentary time were derived for week- and weekend-days and compared across waves via linear multilevel models. We also analysed the date of data collection as a time series, to explore temporal patterns via generalised additive mixed models.

**Results:**

There was no difference in children’s mean MVPA in Wave 2 (weekdays: -2.3 min; 95% CI: -5.9, 1.3 and weekends: 0.6 min; 95% CI: -3.5, 4.6) when compared to the pre-COVID-19 data. Sedentary time remained higher than pre-pandemic by 13.2 min (95% CI:5.3, 21.1) on weekdays. Differences compared to pre-COVID-19 changed over time, with children’s MVPA decreasing over winter, coinciding with COVID-19 outbreaks, and only returning to pre-pandemic levels towards May/June 2022. Parents’ sedentary time and weekday MVPA was similar to pre-COVID-19 levels, with MVPA higher than pre-pandemic by 7.7 min (95% CI: 1.4, 14.0) on weekends.

**Conclusion:**

After an initial drop, children’s MVPA returned to pre-pandemic levels by July 2022, while sedentary time remained higher. Parents’ MVPA remained higher, especially at weekends. The recovery in physical activity is precarious and potentially susceptible to future COVID-19 outbreaks or changes in provision, and so robust measures to protect against future disruptions are needed. Furthermore, many children are still inactive, with only 41% meeting UK physical activity guidelines, and so there is still a need to increase children’s physical activity.

**Supplementary Information:**

The online version contains supplementary material available at 10.1186/s12966-023-01441-1.

## Introduction

Physical activity is important for physical and mental health [1–3]. The World Health Organization and UK Chief Medical Officers recommend that children and young people should engage in an average of an hour of moderate-to-vigorous intensity physical activity (MVPA) per day, accumulated across the week, and that all adults should engage in 150 min or more of MVPA per week [1–3]. In addition, there is some evidence to suggest that elevated sedentary time is associated with increased adiposity and decreased fitness [4, 5], and WHO guidance recommends limiting sedentary behaviour, especially recreational screen time [2]. However, a number of studies have shown that large proportions of children and young people [6, 7] as well as adults [8–10] do not meet these recommendations.

The coronavirus disease 2019 (COVID-19) SARS-Cov-2 pandemic resulted in both acute and longer-term impacts on the physical activity opportunities for all aspects of society. National lockdowns were implemented in many countries, during which traditional opportunities for physical activity such as school sports, swimming and gym sessions were removed, but conversely much of the population were at home for large periods of the day and were encouraged to exercise. Studies conducted during the acute phase of the pandemic reported a general picture of physical activity levels reducing among adults and children [11–14], while sedentary time and screen-viewing increased [11, 15]. Levels of activity were influenced by personal, organisational, environmental and policy factors [16], with less impact in rural settings which offered opportunities for outdoor play [17, 18]. As lockdowns and restrictions are lifted and countries return to “normal” it is unclear how active children and adults are.

Prior to the COVID-19 pandemic, physical activity patterns among children and adults in the UK have been relatively stable. For example, analysis of the Health Survey for England data in 2012 and 2015 reported that patterns of children’s [19] and adults’ [10] physical activity were broadly consistent when measured using the same methods in both years. COVID-19 had an important impact on physical activity with many studies across multiple countries reporting large decreases in the duration and frequency of physical activity in both children and adults [11, 12] during and immediately after the acute phase of the pandemic, with physical activity levels reflective of the level of restrictions in place [20]. It is important to highlight that most of the evidence on the impact of COVID-19 on physical activity was reliant on self-reported physical activity from non-representative convenience samples [11, 12] and as such more information from studies using personal monitoring devices such as accelerometers, and more representative populations, is needed.

We recently added to this evidence base by using a natural experiment design to report on the accelerometer-measured physical activity and sedentary time of 393 Year 6 children (aged 10–11) and their parents/carers who were assessed in Wave 1 of the Active-6 study [21] between May and December 2021. We then compared the physical activity of these participants to 1,296 children and parents/carers, recruited from the same schools three years earlier (pre-pandemic). The analysis showed that on average these children engaged in 7–8 fewer minutes of MVPA per day and 25 extra minutes of sedentary time compared to children pre-pandemic but there was no difference in parental physical activity or sedentary time [21]. These data are consistent with a similar natural experiment study from Central Texas which reported that 8–11-year-old children engaged in around 9 fewer minutes of accelerometer-measured MVPA than in the pre-pandemic comparator [22]. Collectively these studies highlight that the COVID-19 pandemic has resulted in marked impacts on physical activity of children but the impact on adults is uncertain. We do not know if these patterns remained as the acute phase of the pandemic has eased or how patterns of behaviour among children and their parents have changed over time. The goal of this paper is to fill this important gap by reporting on the physical activity and sedentary time of Year 6 children and their parents measured in Wave 2 of the Active-6 study between January and July 2022 and compare levels of physical activity with both the Wave 1 data and the pre-COVID-19 comparator group. We also sought to examine how the difference in physical activity compared to pre-COVID-19 changes over the full duration of the study (14 months) and if there was any evidence of differences by gender or socio-economic position.

## Methods

**Fig. 1:**
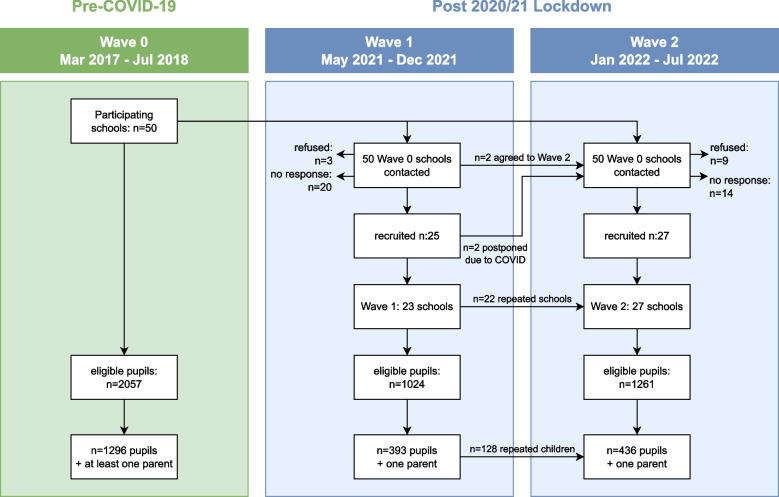
Flow diagram of study participants

Active-6 [21, 23] is a natural experiment using repeated cross-sectional data to assess the acute and longer-term effects of the COVID-19 pandemic on the physical activity of children aged 10–11 years. We compared data from a pre-COVID-19 comparator group to new data collected in two waves between May 2021 and July 2022, after lockdowns and strict COVID-19 restrictions had been lifted. The pre-COVID-19 data are taken from the longitudinal study, B-Proact1v [7], which collected accelerometer and questionnaire data from 10–11-year-old children and at least one parent/carer from 50 schools in and around Bristol, UK between March 2017 and May 2018. In Active-6, we invited the same 50 schools to participate, with all 10–11-year-old children and one parent/carer per family eligible. In both studies, siblings or twins were eligible if they were in the same school year (Year 6 in the UK, aged 10–11 years). Twenty-three schools participated in Wave 1 (May-December 2021), and 27 in Wave 2 (January-July 2022), of which 22 participated in both Waves. At all measurement points, we collected child and parent accelerometer and questionnaire data. Both B-Proact1v and Active-6 received ethical approval from the School of Policy Studies Ethics Committee at the University of Bristol, UK, and parental consent was received for all participants. A total of 1296 child-parent pairs participated pre-COVID-19, with 393 in Wave 1 and 436 in Wave 2, including 128 who participated in both Waves 1 and 2 (Fig. 1).

### Data

Children and one parent/carer per family wore a waist-worn ActiGraph wGT3X-BT accelerometer (Actigraph LLC; Florida, US) during waking hours for five consecutive days, including two weekend days, in the pre-COVID-19 study, and for seven consecutive days in the Active-6 study. Accelerometer data were processed using an open-source R script [24], and analysis was restricted to participants who provided at least two valid weekdays of data for weekday summaries and at least one valid weekend day of data for weekend summaries. We excluded data between midnight and 6am and defined a valid day as at least 500 min of data, excluding intervals of ≥ 60 min of zero counts allowing up to two minutes of interruptions [6]. Mean weekday and weekend minutes of MVPA and sedentary time were derived from 10 s epochs using Evenson population-specific cut-points for children [25], and Troiano cut-points for adults [26]. The average accelerometer wear time was also recorded. Child gender, child date of birth, parent gender, parent age group and the highest education qualification in the household were reported by the parent/carer in a questionnaire (paper or online pre-COVID-19, and online only for Waves 1 and 2). Daily data on lab-confirmed COVID-19 cases between June 2021 and July 2022 for 5–14 year olds in the Southwest region were extracted from UK Government official data for comparison [27].

### Statistical analysis

The analysis was pre-specified in a statistical analysis plan [28], with the primary outcome as average daily child weekday MVPA. Child weekend MVPA and sedentary time, and parental weekday and weekend MVPA and sedentary time were secondary outcomes. Levels of missing data were reported, and descriptive summaries of pupil and parent demographics (age, gender and household education) were reported by wave. Estimates of average child and parent daily physical activity were reported by wave with 95% confidence intervals adjusted for clustering.

To allow for the study design and repeated children in Waves 1 and 2, we used 2-level mixed effects models with repeated measurements nested within children nested within schools, and child and school-level random intercepts. All schools were included in the model regardless of how many waves they participated in. To estimate differences in MVPA for each wave compared to pre-COVID-19, we fitted a linear mixed effects model, with wave as a categorical explanatory variable, and pre-COVID-19 as the reference category. Models were adjusted for accelerometer wear time (in minutes), differences in COVID-19 restrictions at the time of data collection [21], hours of daylight and seasonality, as well as age, gender and highest household education. We did not adjust for child BMI as associations with physical activity are complex [7, 29], especially when looking at differences over time. Seasonality was modelled using separate second order harmonic sine/cosine functions [25] for before and after COVID-19 lockdown, for consistency with Wave 1 results. As more covariates in the regression model will generally increase the standard errors of key parameters, we reduced the number of seasonal variables via a combination of p-values, model fit (Akaike Information Criterion: AIC), variance inflation factors and interpretability to obtain the most parsimonious model. This model estimates a single difference for each wave compared to pre-COVID-19 and is similar to the published Wave 1 analysis [21].

To explore how post-lockdown physical activity changed over the duration of the study, we plotted observed physical activity against time of data collection using a non-parametric loess smoother [26], and compared it to the predicted physical activity for school and time of year, based on the pre-COVID-19 data. Predictions were made using a generalised additive model with random effects for school and a smooth spline for day of year and adjusted for wear time and demographics (age, gender and household education). We also included on the Figure the reported number of COVID-19 cases among 5–14 year olds in the area for reference. We then modelled nonlinear change over time compared to pre-COVID-19, using a generalised additive mixed model (GAMM) [30] which uses splines to model nonlinear associations. We used a smooth nonlinear thin-plate regression spline [31] for the time since the start of Wave 1, and a cyclic cubic spline (to avoid a discontinuity at December/January) for time of year. It is difficult to separate background seasonality from the COVID-19 effect with the data available, as both change over time, and so we repeated this model without the seasonal cyclic spline in a sensitivity analysis. All GAMMs were adjusted for accelerometer wear time, gender, age and household education. We also explored differences by gender and household education by fitting separate change over time for each group. These were compared visually and using AIC to compare model fit [30].

Descriptive summaries were performed in Stata v15 [32] and the linear and generalised additive mixed models were run in R v4.2.0 [33] using the mgcv package v1.8. All model assumptions were checked via visual inspection of the fixed and random residuals. We analysed complete case data for each model rather than using imputation methods, firstly as the majority of missing data is in the outcome and secondly as routine multiple imputation methods are not available for complex statistical models such as the GAMM used here. Note that the mixed effect models used in the paper include data from all schools at all timepoints.

## Results

Child and parent demographics (Table 1) were comparable between Wave 1 and Wave 2. Although children in both post-lockdown waves were more likely to come from households with higher educational qualifications than the pre-COVID-19 sample, other demographics were comparable. Missing data (Additional File: Table S1) varied between 0–28%, with the majority of missing data due to lack of accelerometer data, and similar levels of missing accelerometer data across all three waves, at around 10% for child and 18% for parent weekday physical activity, rising to around 25% for child and parent data at weekends. Table 2 gives estimates of the average MVPA, light physical activity and sedentary time with 95% confidence intervals. Note that while standard errors have been adjusted for clustering, these estimates do not take into account other factors such as using the same schools, seasonality, accelerometer wear time or demographics. Wave 2 estimates of child MVPA were similar to pre-COVID-19 estimates, with slightly higher sedentary time, and 41% met the UK physical activity guidelines in Wave 2, compared to 40% pre-COVID, and 37% in Wave 1. Parent activity was comparable across all waves, with weekend MVPA slightly higher in Wave 2.

**Table 1:** Child and parent demographics by wave

	Pre-COVID-19 Mar 2017-May 2018 *N* = 1296		Wave 1 Jun 2021-Dec 2021 *N* = 393		Wave 2 Jan 2022-Jul 2022 *N* = 436	
**Child age: mean (SD)**	11.0	(0.4)	10.9	(0.4)	11.1	(0.3)
**Child gender: N (%)**						
Male	616	(48%)	198	(50%)	212	(49%)
Female	680	(52%)	183	(49%)	224	(51%)
Other			2	(1%)	0	(0%)
**Parent age: N (%)**						
< 39 yrs	248	(23%)	118	(30%)	121	(28%)
40–44 yrs	414	(39%)	136	(35%)	147	(34%)
45 + yrs	401	(38%)	134	(35%)	161	(38%)
**Parent gender: N (%)**						
Male	294	(27%)	91	(23%)	97	(23%)
Female	794	(73%)	297	(77%)	332	(77%)
Other			1	(< 1%)	0	(0%)
**Parent ethnicity: N (%)**						
White British	944	(87%)	310	(85%)	323	(82%)
White other	57	(5%)	24	(7%)	27	(7%)
Black/African/Caribbean/Asian	56	(5%)	21	(6%)	33	(8%)
Mixed/Other	26	(2%)	8	(2%)	10	(3%)
**Highest household education: N (%)**						
Below University degree^a^	555	(47%)	131	(34%)	162	(38%)
University degree or higher^a^	636	(54%)	257	(66%)	267	(62%)

*SD* Standard deviation

^a^or equivalent qualifications

**Table 2:** Raw estimates of child and parent physical activity by wave

	Pre-COVID-19 Mar 2017-May 2018		Wave 1 Jun 2021-Dec 2021		Wave 2 Jan 2022-Jul 2022	
	Estimate	95% CI	Estimate	95% CI	Estimate	95% CI
**Child**						
Mean weekday MVPA (min/day)	60.3	(57.2, 63.4)	55.9	(52.1, 59.7)	57.8	(54.2, 61.5)
Mean weekday light physical activity (min/day)	209.4	(204.8, 213.9)	197.9	(190.8, 205.1)	199.3	(193.2, 205.3)
Mean weekday sedentary (min/day)	476.8	(470.1, 483.6)	488.4	(477.8, 498.9)	483.6	(475.5, 491.7)
Mean weekend MVPA (min/day)	53.4	(49.8, 57.0)	45.6	(41.8, 49.4)	55.6	(49.9, 61.3)
Mean weekend light physical activity (min/day)	195.2	(190.0, 200.5)	185.0	(179.7, 190.4)	191.3	(182.8, 199.8)
Mean weekend sedentary (min/day)	437.1	(429.3, 444.9)	452.6	(442.7, 462.4)	449.1	(436.9, 461.2)
% meeting UK PA guidelines	40%	(34%, 46%)	37%	(29%, 44%)	41%	(32%, 49%)
**Parent**						
Mean weekday MVPA (min/day)	54.6	(52.2, 57.0)	55.3	(51.5, 59.1)	56.1	(52.0, 60.2)
Mean weekday light physical activity (min/day)	198.5	(193.9, 203.1)	190.7	(184.7, 196.7)	191.7	(183.5, 199.9)
Mean weekday sedentary (min/day)	542.3	(535.4, 549.2)	522.1	(513.4, 530.7)	520.9	(511.4, 530.4)
Mean weekend MVPA (min/day)	46.4	(43.2, 49.6)	49.1	(44.1, 54.0)	53.8	(47.7, 59.8)
Mean weekend light physical activity (min/day)	191.6	(186.8, 196.5)	191.0	(183.8, 198.2)	187.8	(180.8, 194.9)
Mean weekend sedentary (min/day)	486.5	(480.4, 492.6)	487.1	(475.7, 498.6)	477.8	(467.2, 488.4)
% meeting UK PA guidelines	82%	(79%, 86%)	85%	(80%, 89%)	83%	(78%, 88%)

*MVPA* Moderate-to-vigorous physical activity, *PA* Physical activity, *CI* Confidence interval

**Fig. 2:**
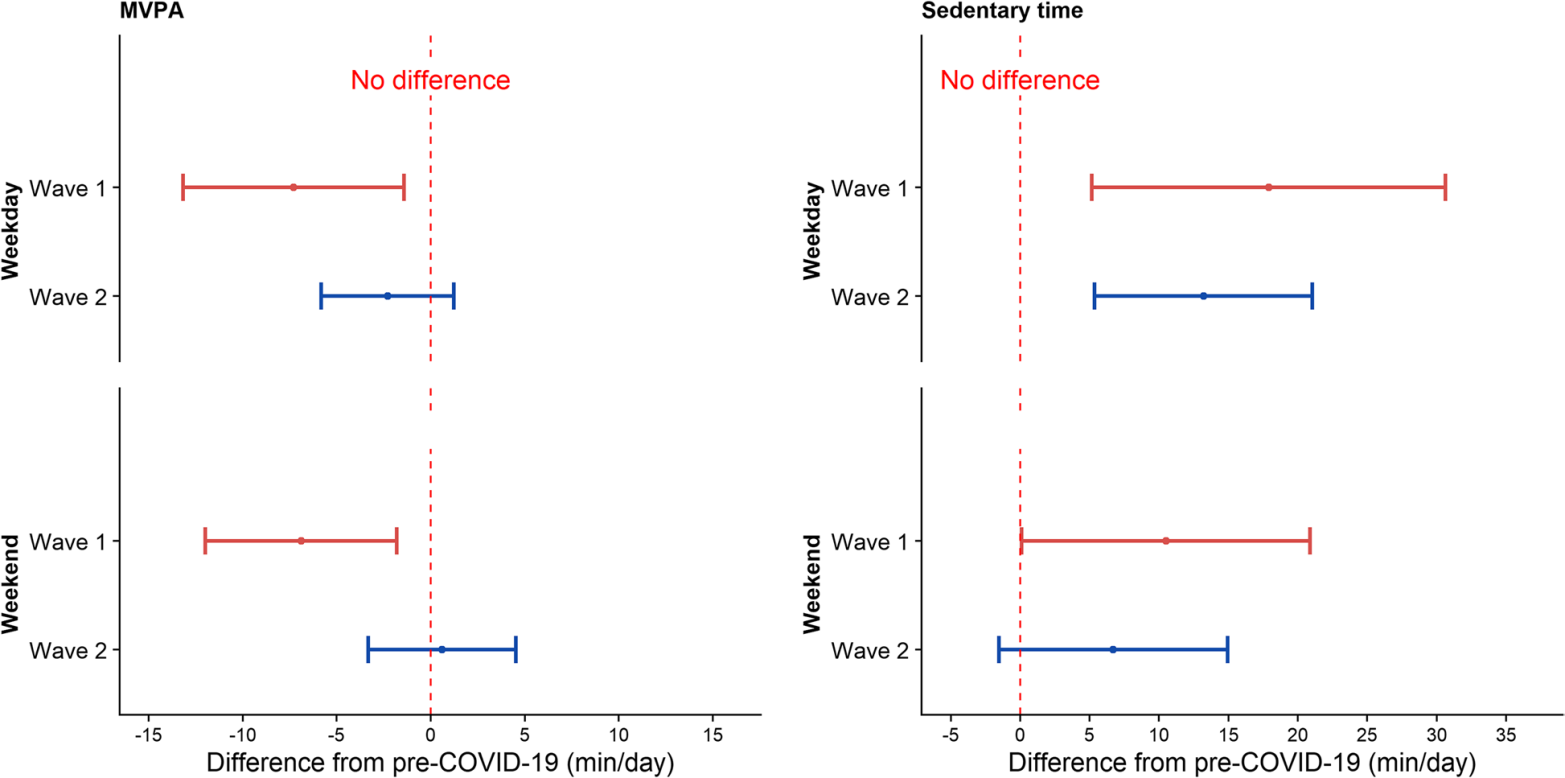
Modelled differences in child MVPA and sedentary time by wave, compared to pre-COVID-19 MVPA = moderate-to-vigorous physical activity Wave 1: June—December 2021 Wave 2: January – July 2022

**Fig. 3:**
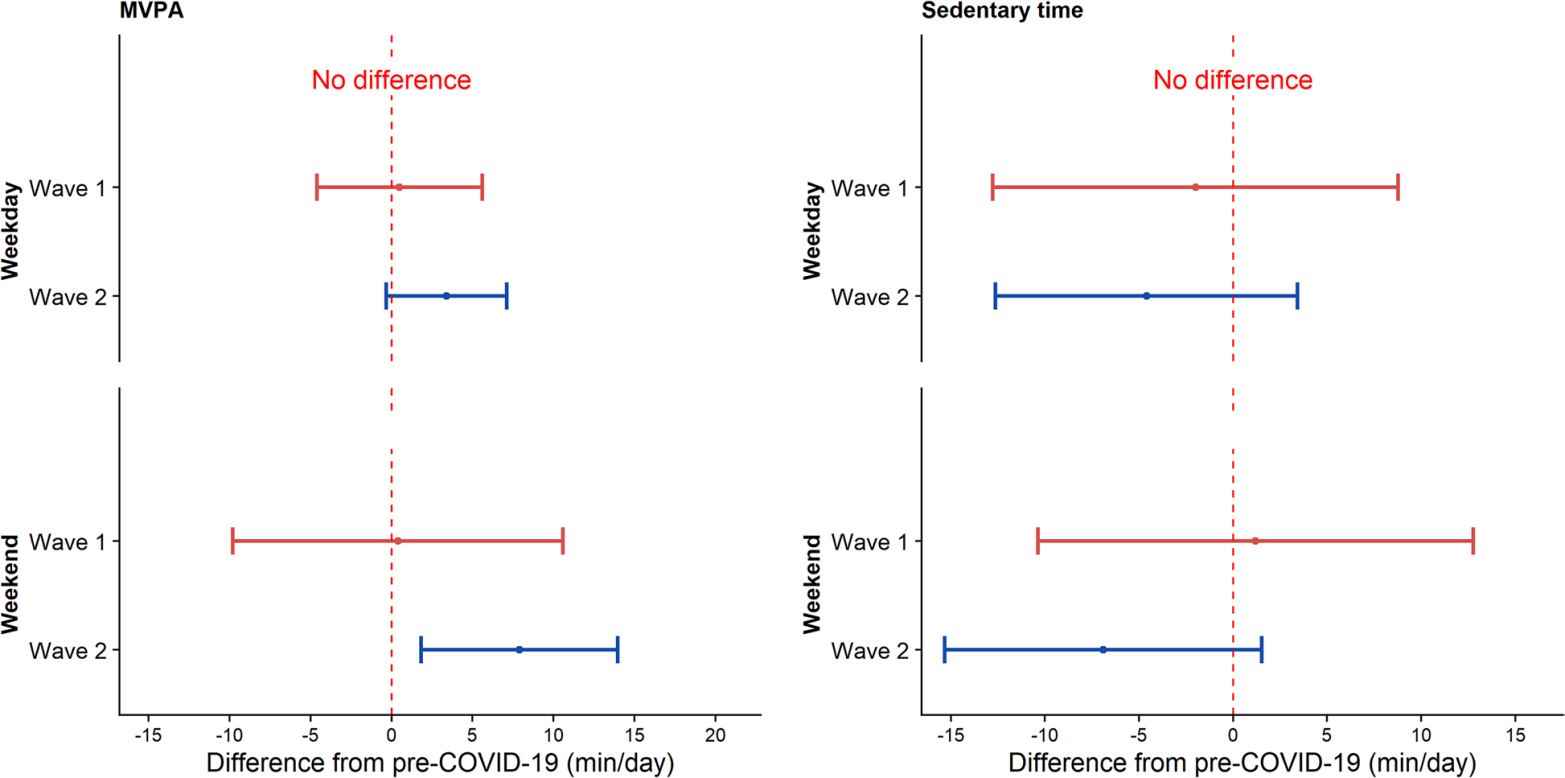
Modelled differences in parent MVPA and sedentary time by wave, compared to pre-COVID-19 MVPA = moderate-to-vigorous physical activity Wave 1: June—December 2021 Wave 2: January – July 2022

Figures 2 and 3 (see also Table 3) shows the modelled estimates of the difference in child and parent MVPA between pre-COVID-19 and Waves 1 and 2, adjusted for accelerometer wear time, daylight hours, seasonality, age, gender and household education. Note that estimates differ very slightly from previously published results [21] due to late-returned data and the use of more data over a longer period of time. Child MVPA was lower than in the pre-COVID-19 comparator group by 7.3 min (95% CI: 1.3 to 13.3) on weekdays in Wave 1, but this reduced to a difference of 2.3 min (95% CI: -1.3 to 5.9) in Wave 2. Similarly, weekend MPVA was lower by 6.9 min (95% CI: 1.7 to 12.1) in Wave 1 but only 0.6 min (95% CI: -4.6 to 3.5) lower in Wave 2, compared to pre-COVID-19. Weekday sedentary time was higher in Wave 1 by 17.9 min (95% CI: 4.9 to 30.8) and remained higher at 13.2 min (95% CI:5.3 to 21.1) in Wave 2, compared to pre-pandemic. Parents’ sedentary time and weekday MVPA were comparable to pre-COVID-19 levels in both post-lockdown waves, with weekend MVPA higher than pre-pandemic levels by 7.7 min (95% CI: 1.4 to 14.0).

**Table 3:** Modelled estimates of differences in child and parent physical activity between Pre-COVID-19 and Post-COVID-19 waves

		Difference between Pre-COVID-19 & Wave 1			Difference between Pre-COVID-19 & Wave 2		
	N	Estimate	95% CI	*p*-value^1^	Estimate	95% CI	*p*-value^1^
**Child**							
weekday MVPA (min/day)	1777	-7.3	(-13.3, -1.3)	0.018	-2.3	(-5.9, 1.3)	0.211
weekend MVPA (min/day)	1493	-6.9	(-12.1, -1.7)	0.010	0.6	(-3.5, 4.6)	0.774
weekday sedentary (min/day)	1777	17.9	(4.9, 30.8)	0.007	13.2	(5.3, 21.1)	0.001
weekend sedentary (min/day)	1493	10.5	(-0.1, 21.1)	0.051	6.7	(-1.6, 15.0)	0.11
**Parent**							
weekday MVPA (min/day)	1581	0.5	(-4.6, 5.6)	0.847	3.4	(-0.4, 7.2)	0.079
weekend MVPA (min/day)	1456	0.4	(-10.1, 10.8)	0.946	7.9	(1.6, 14.2)	0.014
weekday sedentary (min/day)	1581	-2.0	(-13.1, 9.0)	0.713	-4.6	(-12.8, 3.6)	0.265
weekend sedentary (min/day)	1456	1.2	(-10.7, 13.1)	0.843	-6.9	(-15.6, 1.7)	0.115

Pre-COVID-19: Mar 2017-May 2018; Wave 1: Jun 2021-Dec 2021; Wave 2: Jan 2022-Jul 2022

*MVPA* Moderate-to-vigorous physical activity, *CI* Confidence interval

^1^*p*-value for hypothesis test of no difference

**Fig. 4:**
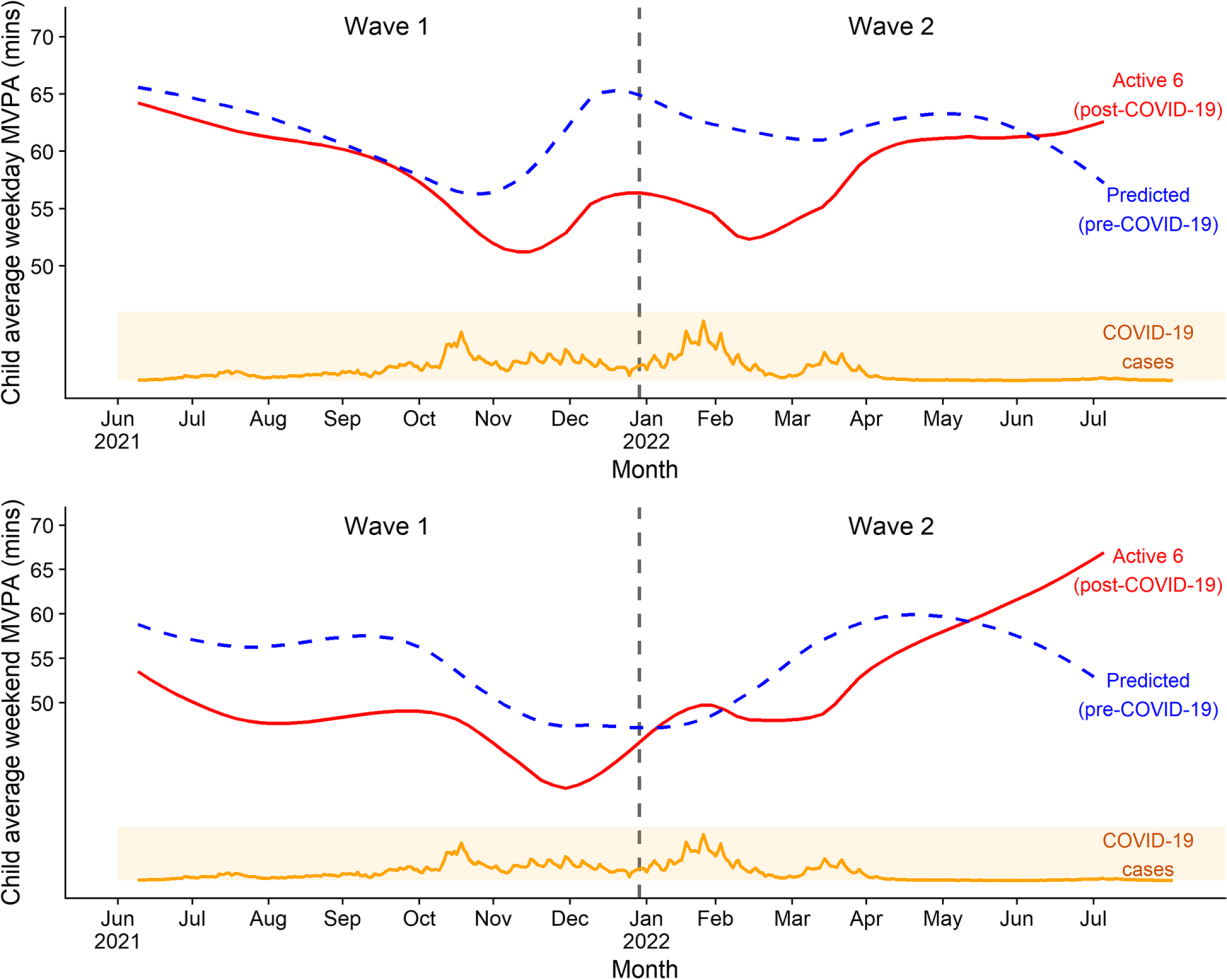
Post-lockdown children’s MVPA with pre-COVID-19 prediction for time of year, and COVID-19 cases MVPA = moderate-to-vigorous physical activity Wave 1: June—December 2021 Wave 2: January – July 2022 COVID-19 cases are for 5–14 year olds in the Southwest region of the UK

**Fig. 5:**
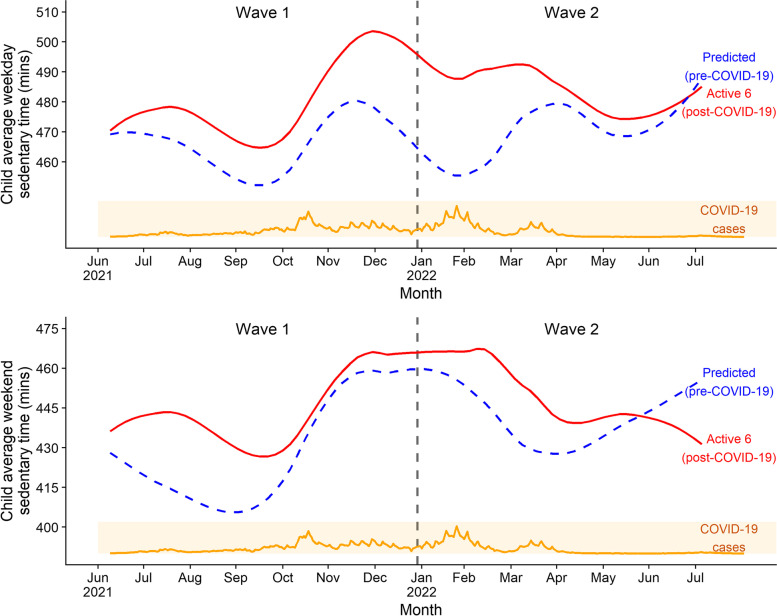
Post-lockdown children’s sedentary time with pre-COVID-19 prediction for time of year, and COVID-19 cases Wave 1: June—December 2021 Wave 2: January – July 2022 COVID-19 cases are for 5–14 year olds in the Southwest region of the UK

Figure 4 shows how children's post-lockdown MVPA changed over the duration of the study, and how this compares to what we would have expected to see based on the pre-COVID-19 data. It also includes the incidence of COVID-19 cases for this age group in the area. Weekday MVPA was slightly lower than expected until October 2021, followed by a larger relative drop over the winter period, and the gap slowly closed as MVPA increased to expected levels by June/July 2022. Weekend MVPA was consistently lower than expected with a small recovery in January/February 2022, and remained lower than expected until May 2022. Both weekday and weekend MVPA levels showed a small drop in February/March 2022 which coincided with an increase in reported COVID-19 cases for this age group in February. Weekday and weekend sedentary time (Fig. 5) was similarly higher than expected throughout most of the study duration.

**Fig. 6:**
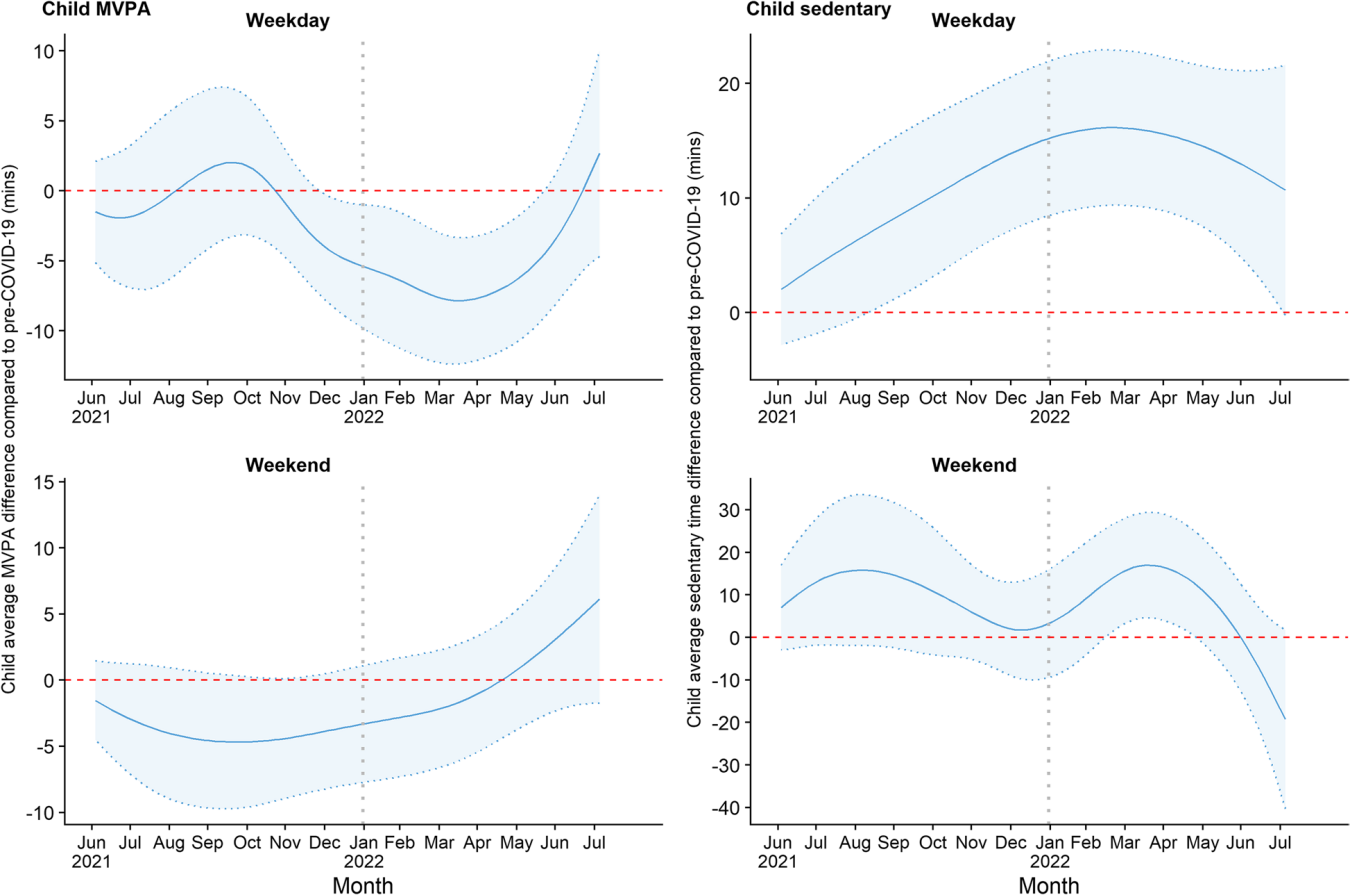
Children’s MVPA and sedentary time modelled change over time with 95% confidence bands MVPA = moderate-to-vigorous physical activity

Figure 6 shows the smoothed estimated difference in children’s MVPA and sedentary time between pre-COVID-19 and post-lockdown based on the GAMM, with a 95% confidence band. Note, this reflects the difference compared to pre-COVID-19, rather than absolute levels of MVPA. Weekday child MVPA dropped below pre-COVID-19 levels from around October/November 2021, reaching a maximum drop of 6.5 min in March/April 2022, and rose to previous levels by the end of the summer term (July 2022). Weekend MVPA was consistently lower between June 2021 and April/May 2022, when it returned to pre-pandemic levels (that is, a difference of 0). Children’s weekday sedentary time was consistently higher throughout the study compared to pre-COVID-19 with a peak of 11 min higher in Jan-March 2022, and was still higher in July 2022 by around 6 min. Weekend sedentary time was likewise higher than pre-pandemic until June/July 2022, with a peak in March/April 2022, at around 15 min higher. Note that although the figure shows an initial peak during August 2021 indicating MVPA levels notably higher than pre-pandemic, no data were collected during the summer holiday period (resulting in the wide confidence bands during this period), so this increase should be treated with caution.

Children’s physical activity had a strong seasonal component, with models that adjusted for seasonality giving better model fit than those without (Additional File: Table S2), which makes estimation of the post-lockdown change over time difficult. Sensitivity analyses which excluded seasonality (Additional File: Figure S1) show broadly similar patterns, but with the high and low differences shifted slightly and larger peaks and troughs. Interaction models showed some gender differences in the trajectories, with girls’ weekday MVPA following a linear difference over time compared to pre-pandemic, in contrast to the drop-and-recovery pattern for boys, and no variation in boys’ weekend sedentary time. However, the AIC did not support separate smooth terms for boys and girls, or for higher and lower education households (Additional File: Figures S2 and S3, Table S3) with wide overlapping confidence bands due to sample sizes.

**Fig. 7:**
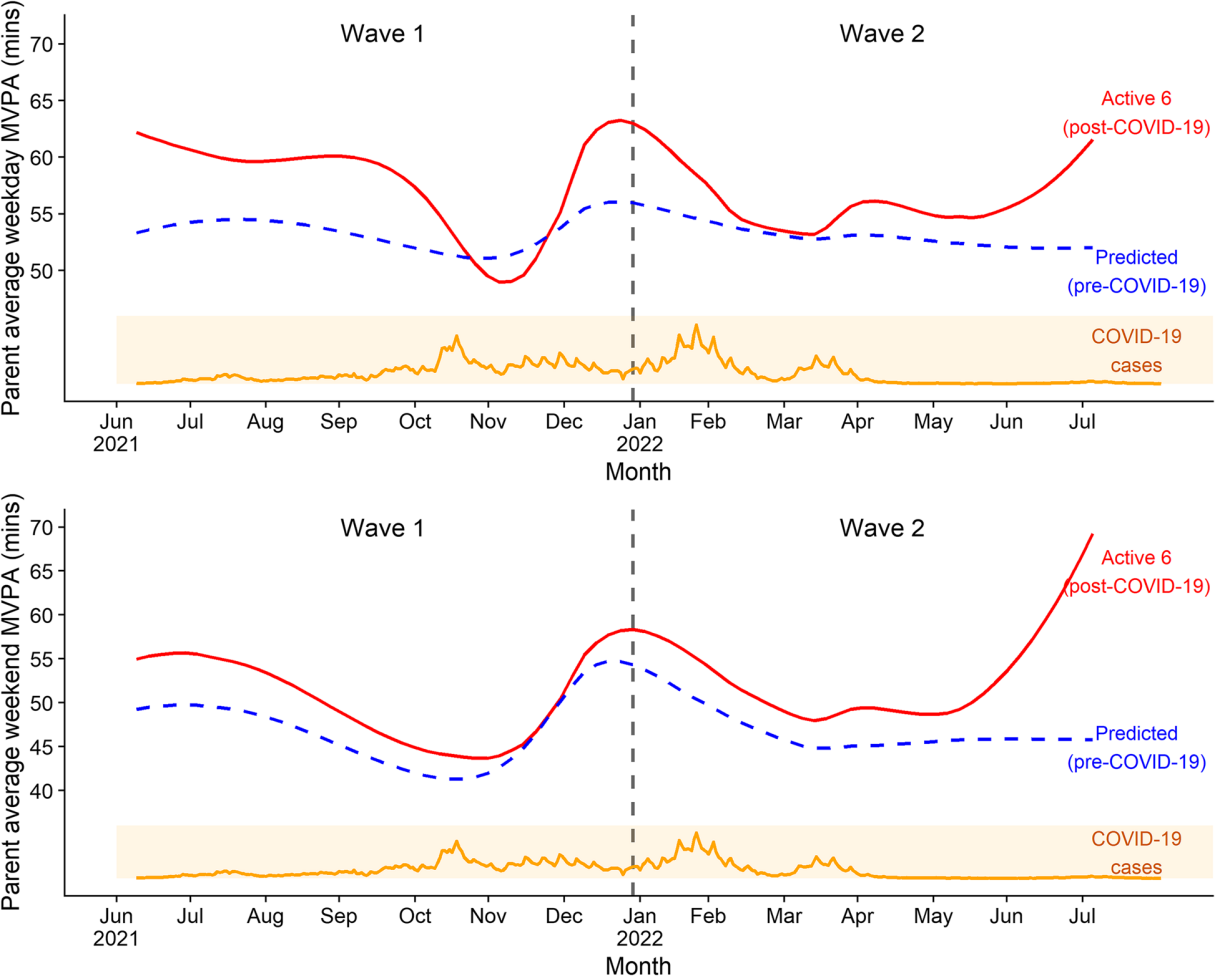
Post-lockdown parents’ MVPA with pre-COVID-19 prediction for time of year, and COVID-19 cases MVPA = moderate-to-vigorous physical activity Wave 1: June—December 2021 Wave 2: January – July 2022 COVID-19 cases are for 5–14 year olds in the Southwest region of the UK

**Fig. 8:**
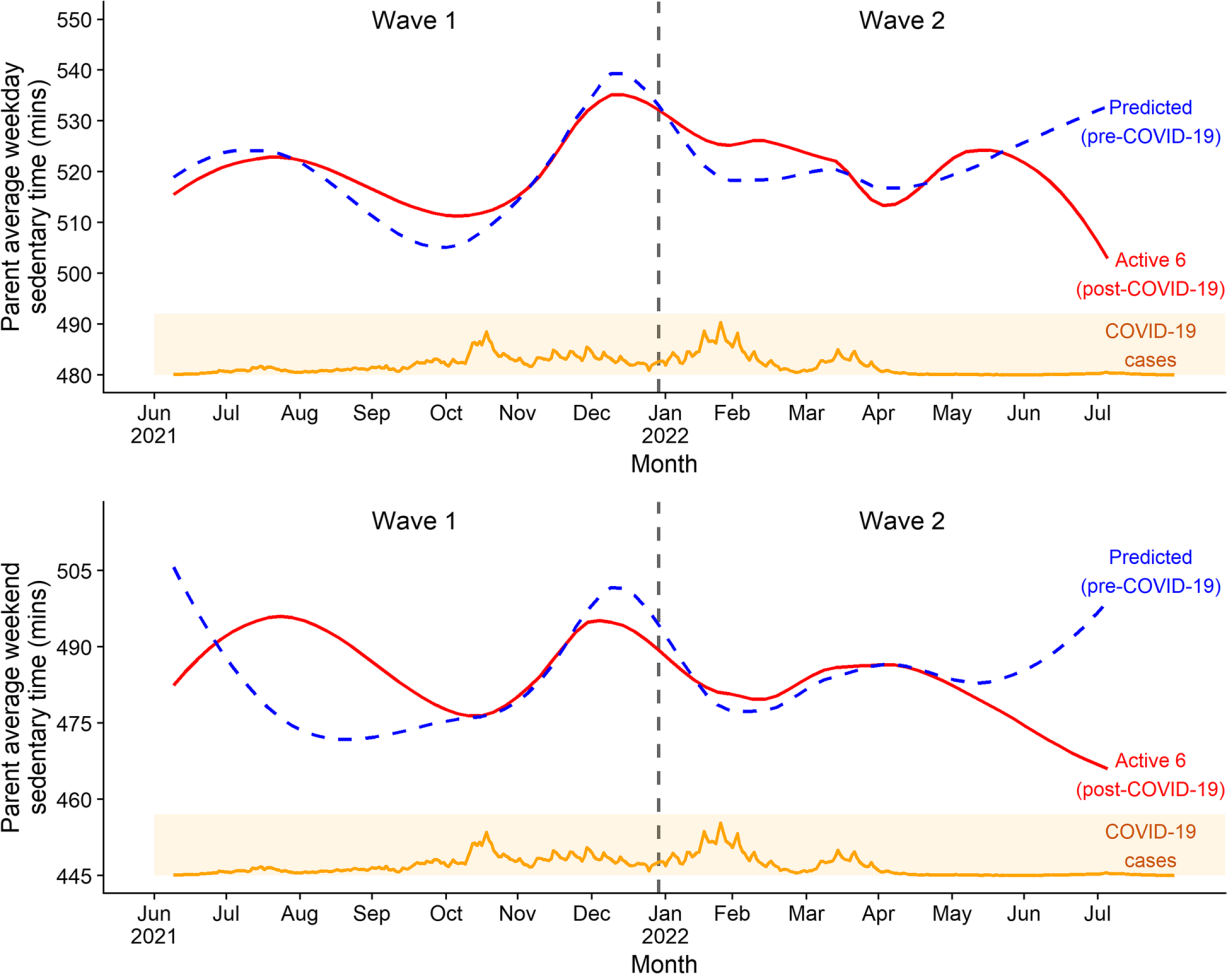
Post-lockdown parents’ sedentary time with pre-COVID-19 prediction for time of year, and COVID-19 cases Wave 1: June—December 2021 Wave 2: January – July 2022 COVID-19 cases are for 5–14 year olds in the Southwest region of the UK

**Fig. 9:**
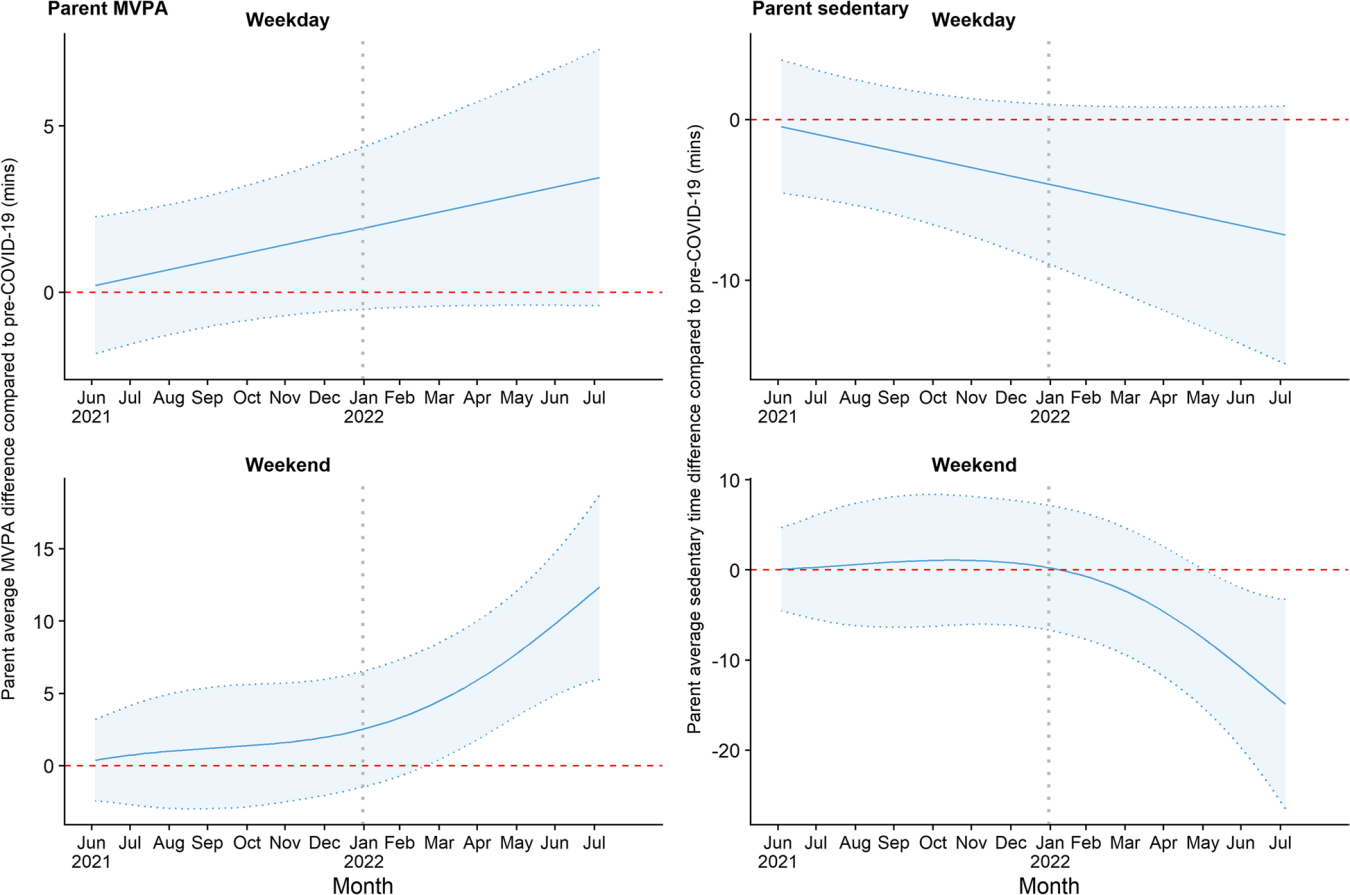
Parents’ MVPA and sedentary time modelled change over time with 95% confidence bands MVPA = moderate-to-vigorous physical activity

The difference in parent physical activity compared to pre-COVID-19 also varied over time. Compared to expected levels based on pre-pandemic data (Figs. 7 and 8) parents’ MVPA was comparable or higher than expected on both weekdays and weekends, with sedentary time broadly comparable. The GAMM models adjusting for seasonality did not produce better model fit (Additional File: Table S2) and so to avoid additional variability in estimates, we reported the model unadjusted for seasonality (Fig. 9). Both weekday and weekend MVPA were higher than pre-pandemic, with the difference in weekday MVPA increasing at a linear rate, while weekend MVPA was initially similar and then increased from February/March 2022 to 10.6 min higher than pre-COVID-19 by July 2022. Parents’ sedentary time was similar to pre-pandemic in 2021, and then dropped from January 2022 to a drop of 8.6 min on weekdays and 15.4 min at weekends by July 2022.

## Discussion

The data reported in this study have shown that although children’s MVPA was around 7–8 min lower than a pre-COVID-19 comparator group between May and December 2021 when social distancing restrictions were initially lifted in the UK, the levels of MVPA were on average close to the comparator group when assessed in a new group about 8 months later on average between January and July 2022. However, weekday sedentary time remained higher than pre-COVID-19 by approximately 13 min per day. There was little difference in parental MVPA in Wave 1 when compared to the reference group with a small increase in Wave 2, especially on weekends. While these findings suggest that patterns of physical activity have more or less returned to pre-pandemic levels, it is important to highlight that at Wave 2 only 41% of children met the hour per day guideline for MVPA while almost 83% of parents met the adult physical activity guidelines. This suggests that there is still an urgent need to find ways to increase the physical activity levels of primary school aged children.

Although the top-level averages suggest that physical activity improved in both children and parents between Wave 1 and Wave 2, these point estimates mask complex temporal patterns. Child average weekday MVPA decreased over winter, a time when physical activity is typically lower [34]. It then continued to decline to around 7 min difference in March–April 2022, before increasing to pre-COVID-19 levels over the summer term (April-July). Weekend MVPA rose slowly from October/November 2021, but was lower than pre-COVID-19 until around April/May 2022. Meanwhile weekday sedentary time increased slowly throughout winter and remained higher than pre-COVID-19 in 2022. The uptick in children’s physical activity only started to occur from May 2022, around 10 months since social distancing restrictions were lifted and all sectors re-opened. This may suggest a number of residual impacts on children’s physical activity. This finding is conceptually consistent with psychological habit theories [35, 36] which emphasise the importance of contextual cues in behavioural execution and maintenance [37]. During the pandemic and the associated lockdowns, many of the contextual cues associated with habitual physical activity for children, such as school, clubs, active travel or playing with friends, were removed for lengthy periods of time, disrupting the automatic processes that underpin the physical activity behaviour [37]. Habits for complex behaviours such as physical activity take time to develop, which might explain why it has taken a year to re-establish pre-pandemic activity levels, especially if underlying habits have changed towards more structured activities and more sedentary behaviours [38]. Further quantitative evidence exploring the daily patterns of activity in children is needed to fully understand this potential shift. In contrast, for adults, while many social contextual cues were also removed during lockdowns, individual habits of physical activity (e.g. running or cycling) may have been more readily adapted and maintained.

The temporal variation in children's post-lockdown MVPA suggests that the observed recovery may be precarious. Not only has it taken nearly a year to fully recover, we note that the lowest levels of MVPA in children occurred when there was a high incidence of COVID-19 in the local area, including a dip in Februrary/March coinciding with a COVID-19 outbreak in this age group. It is not possible to say if this is a causal association, but similar patterns have been observed elsewhere [17] and conversations with school staff reported disruptions to physical activity provision as schools tried to manage infection rates. Provision may have been removed due to staff or pupil illness, or attendance reduced due to illness or in response to concerns about high case numbers. This finding suggests that the ‘recovery’ may be susceptible to future COVID-19 outbreaks and/or reimposed restrictions, even if disruptions to provision are only temporary. More broadly, it suggests that changes to physical activity opportunities may have impacts on both physical activity and sedentary time that can last even when provision reverts to the original levels. Thus, there is a need to think very carefully about any reductions in the provision for children’s physical activity and be mindful that such reductions may have longer term negative effects. There is therefore a need both to protect provision for children’s physical activity in any future outbreaks and to seek to maintain and enhance provision for the long-term physical activity of children and young people.

The importance of this finding may also apply more widely, for example to other respiratory illnesses such as colds and influenza on levels in the local area which may result in further longer-term implications for child physical activity. Similarly, this finding may have implications for the impact of the cost of living and energy crisis in the UK which may affect provision, for example due to cost, closure of facilities, staff retention or reduced attendance. This suggests that special consideration of how to promote physical activity in the current uncertain economic climate, or when there are high levels of (respiratory) illnesses, may be warranted. Equally, future studies may wish to examine the broader impact of community respiratory illnesses on levels of physical activity in children and adults.

We observed gender differences in the way that the COVID-19 pandemic impacted on the physical activity of children, with boys more affected by the COVID-19 lockdowns than girls, although our ability to draw firm conclusions is limited due to high variability in estimates. This could be due to changes in local provision, or it is possible that as boys are more active than girls [6, 7] they have been more affected by change. Previous studies have found both that boys’ activity was more affected than girls’ during lockdowns [12, 13] but also that they increased physical activity more once lockdowns were over [22, 39, 40] and this is consistent with the overall patterns reported here. Further examination of this potential gender difference in other datasets and examination of any potential causes of this difference is warranted, to understand why boys’ physical activity has changed and if there is anything that could be learnt which may help girls to become more physically active.

It is important to consider the seasonality of effects when analysing physical activity data, especially when looking at the effect of the COVID-19 pandemic which itself varies over time. We found that seasonality was stronger in children’s physical activity patterns compared to adults, and that models without adjustment for seasonality tended to overestimate the COVID-19 effect at the peaks and troughs, which tend to coincide with natural highs and lows of physical activity. This is because post-lockdown differences are also seasonal, with larger differences at times of the year where physical activity is typically high or low, and so the seasonal models ascribe some of the COVID-19 change over time to seasonal variation, potentially underestimating the peaks and troughs. In general, this complex interaction between COVID effects and seasonality can cause both under and over-estimation, making it difficult to interpret COVID-19 differences. For example, a Canadian study [20] found that reported reduction in physical activity compared to pre-pandemic was largest during spring when restrictions were at their strictest, with very little difference in summer when restrictions were relaxed. However, the pre-COVID-19 comparison in that study was from the previous winter, a period when physical activity is traditionally at its lowest, and so the impact of even light restrictions may be underestimated. In contrast, a study in Wales [39] found that post-lockdown MVPA in May 2021 was 20 min higher than during lockdown in February 2021, but as physical activity levels are typically higher in spring, this may be an overestimate. A recent meta-analysis of change in physical activity during the COVID-19 pandemic [14] does not include information on what time of year data were collected, and thus the finding that longer study durations were associated with larger reductions could be due to confounding by time of year.

### Strengths and limitations

The major strength of this study is the use of accelerometer-measured physical activity, which took place in the same schools as the pre-pandemic comparator. This minimises any school-level variability which we have previously reported is an important, and often ignored, component of children’s physical activity research [41]. The study is also greatly strengthened by our modelling approach which has enabled us to take account of the temporal patterns in physical activity across the year. The modelling has also enabled us to examine change over time, thereby facilitating the exploration of complex patterns rather than just using arbitrary time periods.

The key limitation of this study is the provision of only one year of data before the COVID-19 pandemic. This means that it is difficult to separate seasonality from post-lockdown change over time, although sensitivity analysis suggests that the impact of this is to over-smooth (that is, estimated temporal patterns are flatter than in truth), and that the true difference may be slightly larger at times of year where physical activity is typically lower. It is also important to highlight that although our data suggest there are likely to be differences in adaptations post-pandemic by gender and socio-economic position the sample is not powered to explicitly test for such differences. Although there were some children who took part in the assessments during both Wave 1 and Wave 2 this was not enough to examine longitudinal change over time (*n* = 128). Finally, Active-6 is a natural experiment which via necessity uses a before-after design, where the only available controls are historical. As such it is possible that differences may be due to other factors rather than COVID-19, especially if these differ over time. Our analysis takes into account seasonal differences, but we cannot rule out longer-term secular changes, although pre-pandemic data have suggested that children’s MVPA is relatively stable over time. Differences could also be due to participant characteristics, such as activity levels, although we do adjust for differences in age, gender and socioeconomic position.

### Implications

The main implication of this work is that despite the recovery to pre-pandemic levels, still only 41% percent of children met the public health guidance, and children’s sedentary time remains higher than pre-pandemic. We thus need to find new ways to increase children’s physical activity and manage sedentary behaviours. In addition, we need to identify how schools and wider aspects of the community can increase provision that children want to use, for example, increased opportunities for children to sample new activities and subsidisation of programs, and ensure this provision is robust to temporary disrputions. Similarly, we need to help families to develop strategies to manage sedentary behaviours. For parents, the findings suggest that many parents are more active than pre-pandemic comparators so we need to understand the causes of the difference between parents and their children and how that can be used to find new ways to promote physical activity.

## Conclusions

The COVID-19 pandemic has affected children’s physical activity in both the short and longer term, with the effect changing over time. Levels of MVPA have returned to pre-pandemic levels after about a year since most restrictions were lifted, while sedentary time remains higher than before. However, this recovery appears precarious and may be susceptible to future outbreaks of COVID-19 or other pandemics or changes in physical activity provision, and so robust measures to protect against future disruptions are needed. Furthermore, even with the recovery, many children are still inactive, with only 41% meeting UK physical activity guidelines, and so there is still a need to increase physical activity, especially amongst those who are typically less active. Further monitoring of physical activity patterns is needed, as well as new approaches to increase children’s physical activity.

## Supplementary Information

**Additional file 1: Additional tables and figures.**

## Data Availability

As the Active-6 project is still ongoing, data are not currently available. Data will be made available in the University of Bristol’s data repository at the end of the project (https://data.bris.ac.uk/data/).

## Abbreviations

AIC: Akaike Information Criterion
COVID-19: Coronavirus disease 2019
GAMM: Generalised additive mixed model
MVPA: Moderate to vigorous physical activity

## References

[1] Bull FC, Al-Ansari SS, Biddle S, Borodulin K, Buman MP, Cardon G, Carty C, Chaput JP, Chastin S, Chou R (2020). World Health Organization 2020 guidelines on physical activity and sedentary behaviour. Br J Sports Med.

[2] Chaput JP, Willumsen J, Bull F, Chou R, Ekelund U, Firth J, Jago R, Ortega FB, Katzmarzyk PT (2020). 2020 WHO guidelines on physical activity and sedentary behaviour for children and adolescents aged 5–17 years: summary of the evidence. Int J Behav Nutr Phys Act.

[3] UK Chief Medical Officers: UK Chief Medical Officers’ Physical Activity Guidelines. London: Department of Health and Social Care; 2019.

[4] Rey-Lopez JP, Vicente-Rodriguez G, Biosca M, Moreno LA (2008). Sedentary behaviour and obesity development in children and adolescents. Nutr Metab Cardiovasc Dis.

[5] Chinapaw MJ, Proper KI, Brug J, van Mechelen W, Singh AS (2011). Relationship between young peoples' sedentary behaviour and biomedical health indicators: a systematic review of prospective studies. Obes Rev.

[6] Cooper AR, Goodman A, Page AS, Sherar LB, Esliger DW, van Sluijs EM, Andersen LB, Anderssen S, Cardon G, Davey R (2015). Objectively measured physical activity and sedentary time in youth: the International children's accelerometry database (ICAD). Int J Behav Nutr Phys Act.

[7] Jago R, Salway R, Emm-Collison L, Sebire SJ, Thompson JL, Lawlor DA (2020). Association of BMI category with change in children's physical activity between ages 6 and 11 years: a longitudinal study. Int J Obes (Lond).

[8] Du Y, Liu B, Sun Y, Snetselaar LG, Wallace RB, Bao W (2019). Trends in Adherence to the Physical Activity Guidelines for Americans for Aerobic Activity and Time Spent on Sedentary Behavior Among US Adults, 2007 to 2016. JAMA Netw Open.

[9] Arias-Palencia NM, Solera-Martinez M, Gracia-Marco L, Silva P, Martinez-Vizcaino V, Canete-Garcia-Prieto J, Sanchez-Lopez M (2015). Levels and Patterns of Objectively Assessed Physical Activity and Compliance with Different Public Health Guidelines in University Students. PLoS ONE.

[10] NHS Digital: Health Survey for England 2016: Physical activity in adults. London: Health and Social Care Information Centre; 2017.

[11] Stockwell S, Trott M, Tully M, Shin J, Barnett Y, Butler L, McDermott D, Schuch F, Smith L. Changes in physical activity and sedentary behaviours from before to during the COVID-19 pandemic lockdown: a systematic review. BMJ Open Sport Exerc Med. 2021;7(1):e000960. 10.1136/bmjsem-2020-000960 PMC7852071 34192010

[12] Rossi L, Behme N, Breuer C. Physical Activity of Children and Adolescents during the COVID-19 Pandemic-A Scoping Review. Int J Environ Res Public Health. 2021;18(21):11440. 10.3390/ijerph182111440 PMC8583307 34769956

[13] Sport England: Active Lives Children and Young People Survey Coronavirus (Covid-19) Report: Mid-May to late-July 2020 (the summer term). UK: Sport England; 2021.

[14] Neville RD, Lakes KD, Hopkins WG, Tarantino G, Draper CE, Beck R, Madigan S. Global Changes in Child and Adolescent Physical Activity During the COVID-19 Pandemic: A Systematic Review and Meta-analysis. JAMA Pediatr. 2022;176(9):886-94. 10.1001/jamapediatrics.2022.2313 PMC9274449 35816330

[15] Burkart S, Parker H, Weaver RG, Beets MW, Jones A, Adams EL, Chaput JP, Armstrong B (2022). Impact of the COVID-19 pandemic on elementary schoolers' physical activity, sleep, screen time and diet: A quasi-experimental interrupted time series study. Pediatr Obes.

[16] Richards AB, Minou M, Sheldrick MP, Swindell N, Griffiths LJ, Hudson J, Stratton G. A Socioecological Perspective of How Physical Activity and Sedentary Behaviour at Home Changed during the First Lockdown of COVID-19 Restrictions: The HomeSPACE Project. Int J Environ Res Public Health. 2022;19(9):5070. 10.3390/ijerph19095070 PMC9105670 35564463

[17] Caldwell HAT, Faulkner G, Tremblay MS, Rhodes RE, de Lannoy L, Kirk SFL, Rehman L, Moore SA (2022). Regional differences in movement behaviours of children and youth during the second wave of the COVID-19 pandemic in Canada: follow-up from a national study. Can J Public Health.

[18] Kellstedt DK, Essay AM, Schenkelberg MA, Rosen MS, Von Seggern MJ, Idoate R, Welk GJ, Rosenkranz RR, Dzewaltowski DA. COVID-19 pandemic and changes in children’s physical activity in a rural US community: a mixed methods study. BMJ Open. 2022;12(10):e062987. 10.1136/bmjopen-2022-062987 PMC9620527 36302579

[19] Sims J, Milton K, Foster C, Scarborough P (2022). A profile of children's physical activity data from the 2012 and 2015 health survey for England. BMC Public Health.

[20] Dubuc MM, Berrigan F, Goudreault M, Beaudoin S, Turcotte S. COVID-19 Impact on Adolescent 24 h Movement Behaviors. Int J Environ Res Public Health. 2021;18(17):9256. 10.3390/ijerph18179256 PMC8431636 34501845

[21] Salway R, Foster C, de Vocht F, Tibbitts B, Emm-Collison L, House D, Williams JG, Breheny K, Reid T, Walker R (2022). Accelerometer-measured physical activity and sedentary time among children and their parents in the UK before and after COVID-19 lockdowns: a natural experiment. Int J Behav Nutr Phys Act.

[22] Ganzar LA, Salvo D, Burford K, Zhang Y, Kohl HW, Hoelscher DM. Longitudinal changes in objectively-measured physical activity and sedentary time among school-age children in Central Texas, US during the COVID-19 pandemic. Int J Behav Nutr Phys Act. 2022;19(1):56. 10.1186/s12966-022-01299-9 PMC9117593 35590329

[23] Protocol for Assessing the impact of COVID-19 on the physical activity of Year 6 children and their parents: Identifying scalable actions to mitigate adverse impacts & provide rapid evidence to policy makers (ACTIVE-6). https://fundingawards.nihr.ac.uk/award/NIHR131847.

[24] Salway R. Accelerometer Processing Code. 2023. 10.17605/OSF.IO/Y8MWU.

[25] Evenson KR, Catellier DJ, Gill K, Ondrak KS, McMurray RG. Calibration of two objective measures of physical activity for children. J Sports Sci. 2008;26(14):1557-65. 10.1080/02640410802334196 18949660

[26] Troiano RP, Berrigan D, Dodd KW, Masse LC, Tilert T, McDowell M. Physical activity in the United States measured by accelerometer. Medicine and science in sports and exercise. 2008;40(1):181-8. 10.1249/mss.0b013e31815a51b3 18091006

[27] GOV.UK Coronavirus (COVID-19) in the UK. https://coronavirus.data.gov.uk/details/download.

[28] Salway R, Jago R, Tibbitts B. The ACTIVE-6 project: detailed statistical analysis plan: Assessing the impact of COVID-19 on the physical activity of Year 6 children and their parents: Identifying scalable actions to mitigate adverse impacts and provide rapid evidence to policy makers. Bristol: University of Bristol; 2021.

[29] Richmond RC, Davey Smith G, Ness AR, den Hoed M, McMahon G, Timpson NJ (2014). Assessing causality in the association between child adiposity and physical activity levels: a Mendelian randomization analysis. PLoS Med.

[30] Wood S. Generalized additive models: an introduction with R. 2nd ed. Chapman and Hall/CRC; 2017.

[31] Wood SN (2003). Thin-plate regression splines. J Roy Stat Soc B.

[32] StataCorp. Stata Statistical Software: Release 15. College Station: StataCorp LLC; 2017.

[33] R Core Team. R: A Language and Environment for Statistical Computing. Vienna: R Foundation for Statistical Computing; 2021.

[34] Atkin AJ, Sharp SJ, Harrison F, Brage S, Van Sluijs EM (2016). Seasonal Variation in Children's Physical Activity and Sedentary Time. Med Sci Sports Exerc.

[35] Wood W, Runger D (2016). Psychology of habit. Annu Rev Psychol.

[36] Gardner B (2015). A review and analysis of the use of 'habit' in understanding, predicting and influencing health-related behaviour. Health Psychol Rev.

[37] Hagger MS (2019). Habit and physical activity: Theoretical advances, practical implications, and agenda for future research. Psychol Sport Exerc.

[38] Walker R, House D, Emm-Collison L, Salway R, Tibbitts B, Sansum K, Reid T, Breheny K, Churchward S, Williams JG (2022). A multi-perspective qualitative exploration of the reasons for changes in the physical activity among 10–11-year-old children following the easing of the COVID-19 lockdown in the UK in 2021. Int J Behav Nutr Phys Act.

[39] Hurter L, McNarry M, Stratton G, Mackintosh K. Back to school after lockdown: The effect of COVID-19 restrictions on children's device-based physical activity metrics. J Sport Health Sci. 2022;11(4):530-6. 10.1016/j.jshs.2022.01.009 PMC8802675 35092856

[40] Dallolio L, Marini S, Masini A, Toselli S, Stagni R, Bisi MC, Gori D, Tessari A, Sansavini A, Lanari M (2022). The impact of COVID-19 on physical activity behaviour in Italian primary school children: a comparison before and during pandemic considering gender differences. BMC Public Health.

[41] Salway R, Emm-Collison L, Sebire SJ, Thompson JL, Lawlor DA, Jago R. A Multilevel Analysis of Neighbourhood, School, Friend and Individual-Level Variation in Primary School Children's Physical Activity. Int J Environ Res Public Health. 2019;16(24):4889. 10.3390/ijerph16244889 PMC6950546 31817182

