# Supplementary material for: Short and medium-term effects of the COVID-19 lockdowns on child and parent accelerometer-measured physical activity and sedentary time: a natural experiment
Source: Int J Behav Nutr Phys Act. 2023 Apr 27;20:42. doi: 10.1186/s12966-023-01441-1 (PMC10132917; doi:10.1186/s12966-023-01441-1)
Supplement: Supplementary file 1 — Additional file 1: Additional tables and figures. [file 12966_2023_1441_MOESM1_ESM.docx]

# **Supplementary Material**

## **Short and medium-term effects of the COVID-19 lockdowns on child and parent accelerometer-measured physical activity and sedentary time: a natural experiment**

Russell Jago PhD^1,2, 3,4*^, Ruth Salway^1^, Danielle House^1^, Robert Walker^1^, Lydia Emm-Collison^1^, Kate Sansum^1^, Katie Breheny^2^, Tom Reid^1,2^, Sarah Churchward^5^, Joanna G Williams^2, 6^, Charlie Foster^1^, William Hollingworth^2, 3^, Frank de Vocht^2, 3^

**Additional Figures**

Figure S1: Children’s MVPA and sedentary time modelled change over time, with no seasonality adjustment

Figure S2: Children’s MVPA and sedentary time modelled change over time, with interaction by child gender

Figure S3: Children’s MVPA and sedentary time modelled change over time, with interaction by highest household education

**Additional Tables**

Table S1: Missing data

Table S2 Further model details and model fit for GAMM change over time models.

Table S3 Further model details and model fit for GAMM change over time models with interactions for gender and household education.

Figure S1: Children’s MVPA and sedentary time modelled change over time, with no seasonality adjustment


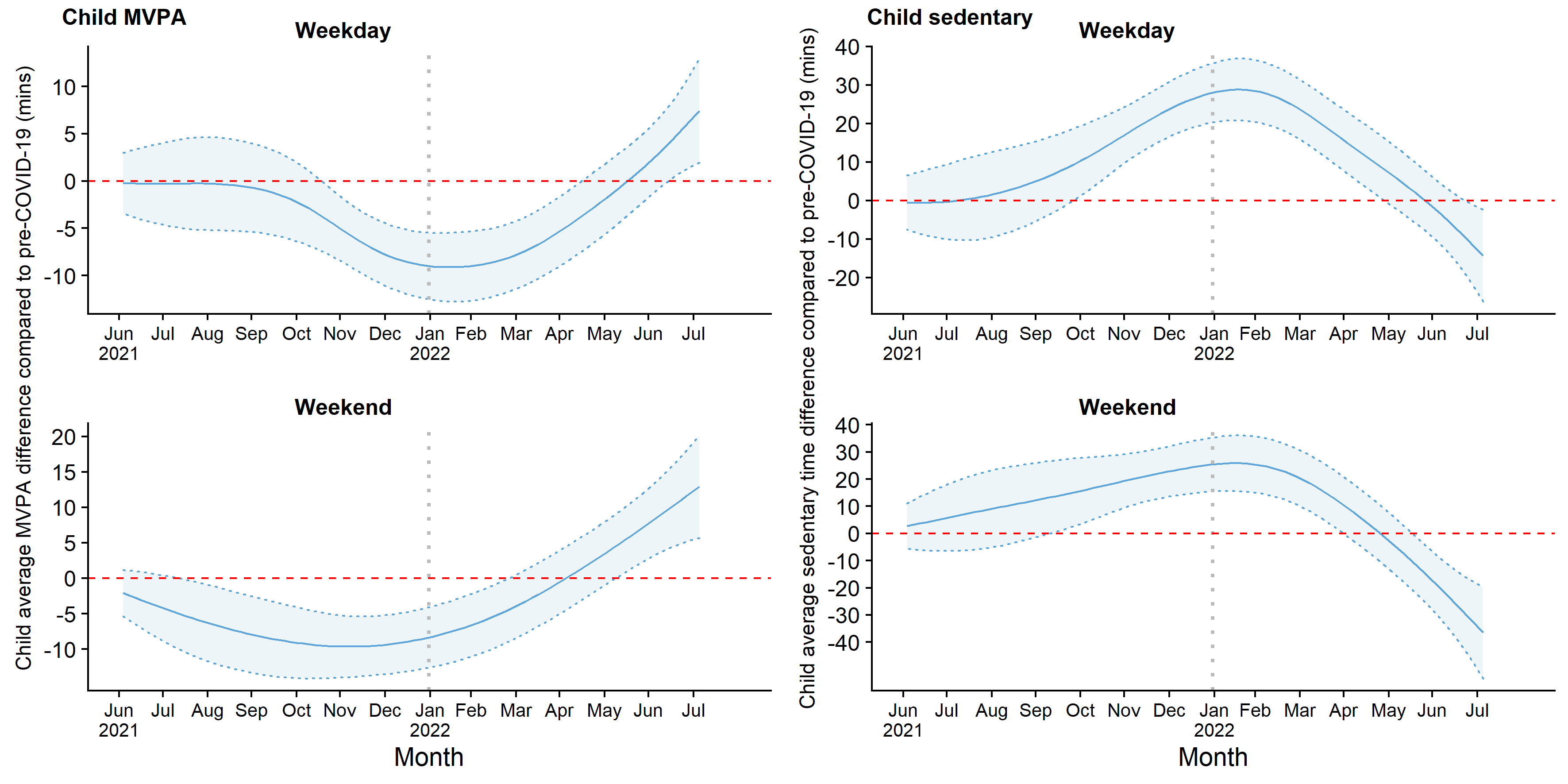


MVPA=moderate-to-vigorous physical activity

Shaded area is 95% confidence band

Figure S2: Children’s MVPA and sedentary time modelled change over time, with interaction by child gender


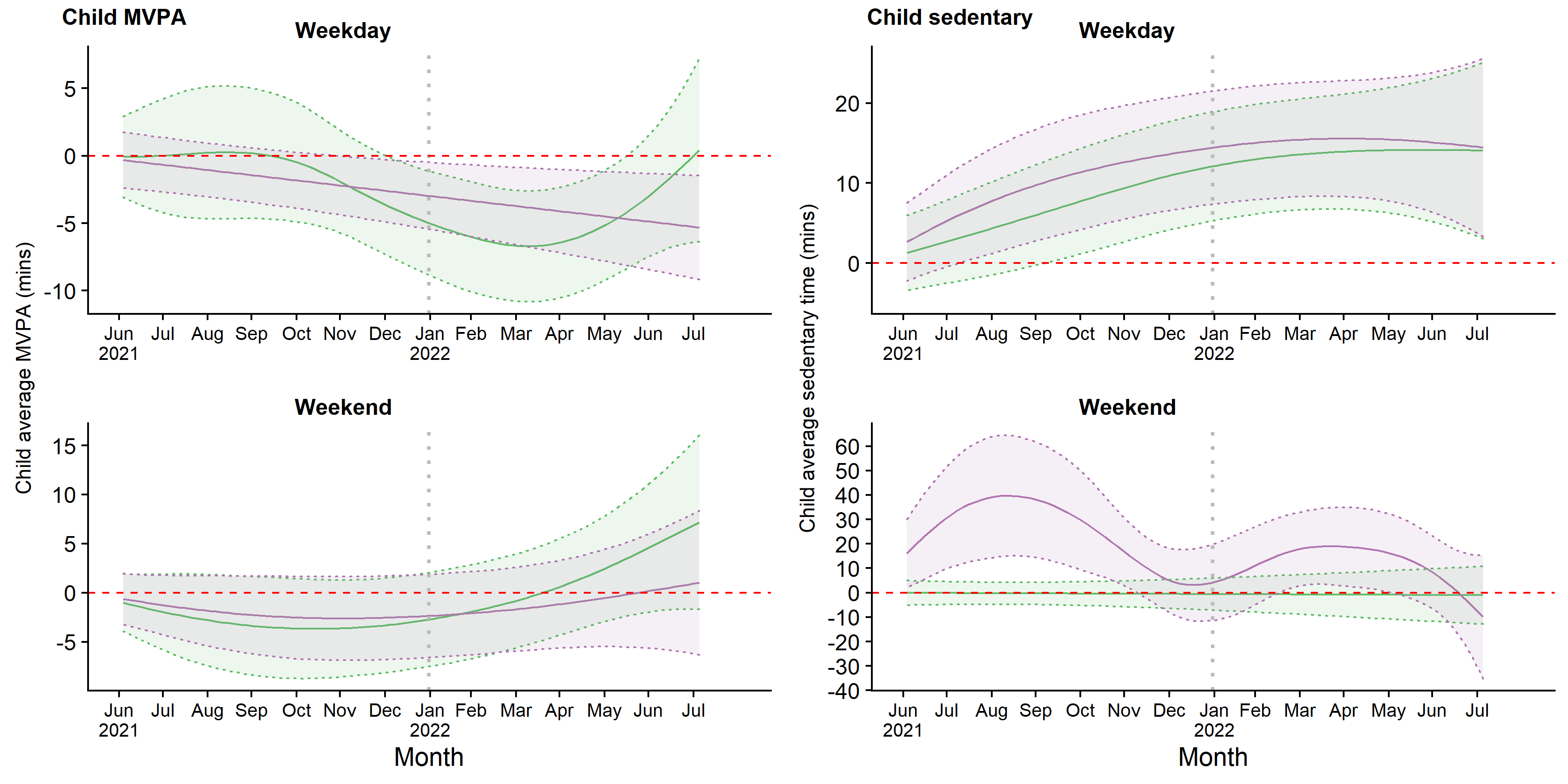


green=boys; purple=girls

MVPA=moderate-to-vigorous physical activity

Shaded areas are 95% confidence bands

Figure S3: Children’s MVPA and sedentary time modelled change over time, with interaction by highest household education


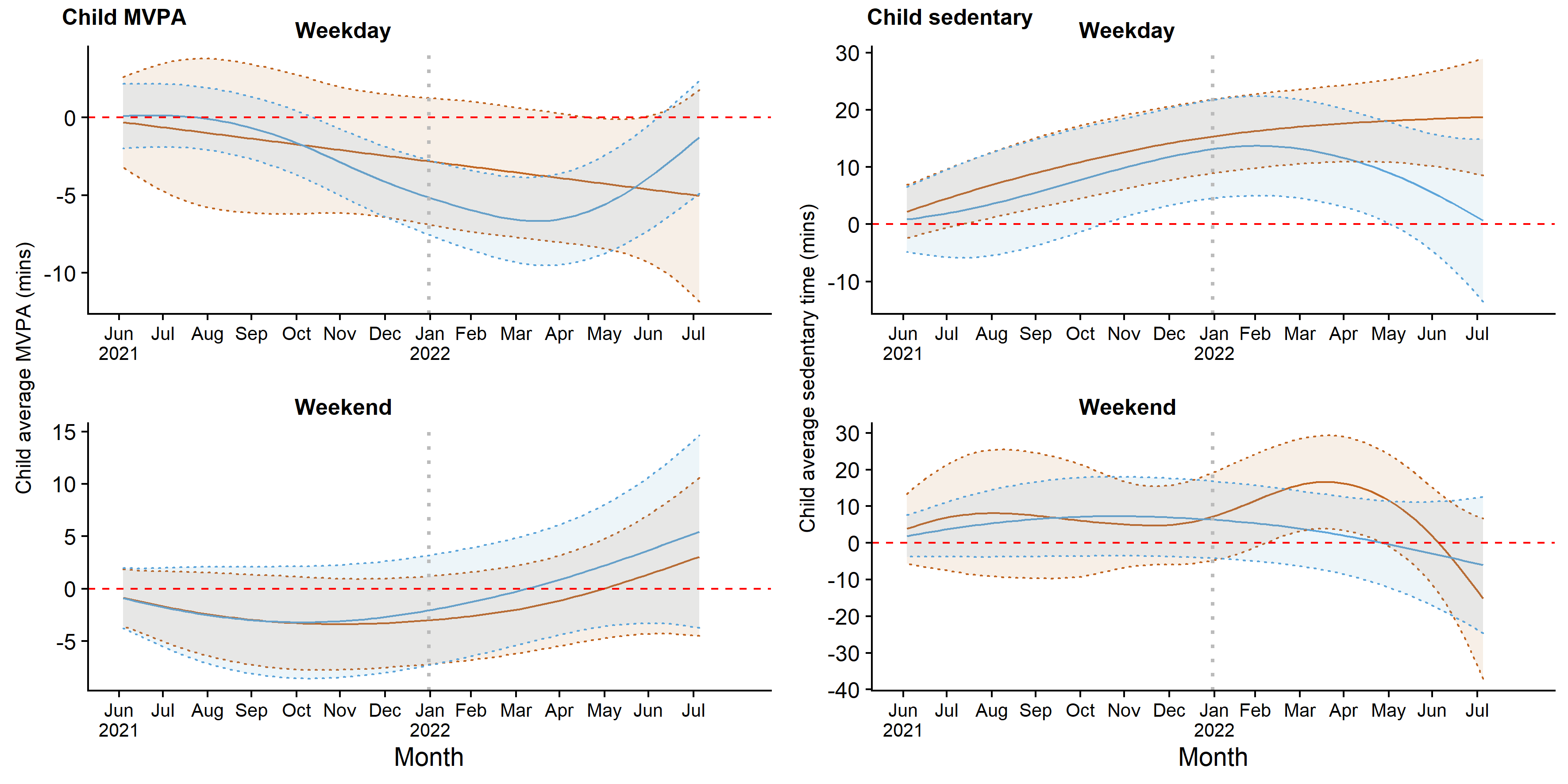


blue= highest household education is up to A level or equivalent qualification at age 18); orange=highest household education is degree or higher

MVPA=moderate-to-vigorous physical activity

Shaded areas are 95% confidence bands

Table S1: Missing data

|  | Wave 0  Mar 2017-May 2018  N=1296 | | Wave 1  Jun 2021-Dec 2021  N=393 | | Wave 2  Jan 2022-Jul 2022  N=463 | |
| --- | --- | --- | --- | --- | --- | --- |
|  | N | % | N | % | N | % |
| **Child** |  |  |  |  |  |  |
| Age | 0 | 0% | 0 | 0% | 0 |  |
| Gender | 0 | 0% | 0 | 0% | 0 |  |
| Weekday accelerometer data | 173 | 13% | 37 | 9% | 42 | 10% |
| Weekend accelerometer data | 332 | 26% | 102 | 25% | 119 | 27% |
| **Parent** |  |  |  |  |  |  |
| Age | 233 | 18% | 5 | 1% | 7 | 2% |
| Gender | 208 | 16% | 5 | 1% | 7 | 2% |
| Household education | 105 | 8% | 5 | 1% | 7 | 2% |
| Ethnicity | 213 | 16% | 30 | 8% | 43 | 10% |
| Weekday accelerometer data | 270 | 21% | 62 | 16% | 75 | 17% |
| Weekend accelerometer data | 313 | 24% | 99 | 25% | 124 | 28% |

Table S2: Model details and model fit for GAMM change over time models.

|  | MVPA | | | | Sedentary time | | | |
| --- | --- | --- | --- | --- | --- | --- | --- | --- |
|  | Estimated  df | p-value^1^ | AIC^2^ | Change in AIC^2^ | Estimated df | p-value^1^ | AIC^2^ | Change in AIC^2^ |
| **Child – not adjusted for seasonality** | | | | | | | | |
| Weekday | 4.1 | <0.001 | 15642 |  | 4.3 | <0.001 | 18484 |  |
| Weekend | 2.9 | <0.001 | 14324 |  | 3.7 | <0.001 | 16459 |  |
| **Child – adjusted for seasonality** | | | | | | | | |
| Weekday | 4.8 | 0.001 | 15627 | -15 | 2.2 | <0.001 | 18465 | -19 |
| Weekend | 2.4 | 0.111 | 14302 | -22 | 4.3 | 0.013 | 16420 | -39 |
| **Parent - not adjusted for seasonality** | | | | | | | | |
| Weekday | 1.0 | 0.103 | 14883 |  | 1.5 | 0.108 | 17335 |  |
| weekend | 2.1 | <0.001 | 14069 |  | 2.1 | 0.056 | 15968 |  |
| **Parent - adjusted for seasonality** | | | | | | | | |
| Weekday | 1.0 | 0.140 | 14884 | 1 | 1.0 | 0.178 | 17337 | 2 |
| weekend | 1.0 | 0.001 | 14070 | 1 | 2.0 | 0.086 | 15970 | 2 |

GAMM=Generalised Additive Mixed Model; MVPA=moderate-to-vigorous physical activity; df=degrees of freedom; AIC=Akaike Information Criterion

^1^ p-value for a hypothesis test of linearity i.e. estimated df=1

^2^ Change reported for seasonality vs no seasonality adjustment. Lower AIC indicates better model fit. Change in AIC of 5 or less generally considered to indicate no improvement in model fit.

Table S3 Further model details and model fit for child GAMM change over time models with interactions for gender and household education.

|  | MVPA | | | | Sedentary time | | | |
| --- | --- | --- | --- | --- | --- | --- | --- | --- |
|  | Est.  df | P-value^1^ | AIC^2^ | Change in AIC^2^ | Est.  df | P-value^1^ | AIC^2^ | Change in AIC^2^ |
| **Gender** | | | | | | | | |
| **Weekday** |  |  |  |  |  |  |  |  |
| Boys | 3.3 |  |  |  | 1.9 |  |  |  |
| Girls | 1.0 | 0.893 | 15630 | 3 | 1.9 | 0.945 | 18468 | 3 |
| **Weekend** |  |  |  |  |  |  |  |  |
| Boys | 2.0 |  |  |  | 1.0 |  |  |  |
| Girls | 1.5 | 0.592 | 14305 | 3 | 5.3 | 0.202 | 16416 | -4 |
| **Household education** | | | | | | | | |
| **Weekday** |  |  |  |  |  |  |  |  |
| No degree | 2.8 |  |  |  | 2.4 |  |  |  |
| Degree | 1.0 | 0.686 | 15631 | 4 | 1.9 | 0.104 | 18464 | -1 |
| **Weekend** |  |  |  |  |  |  |  |  |
| No University degree | 1.7 |  |  |  | 1.7 |  |  |  |
| University degree | 1.9 | 0.677 | 14306 | 4 | 3.8 | 0.513 | 16419 | -1 |

GAMM=Generalised Additive Mixed Model; MVPA=moderate-to-vigorous physical activity; df=degrees of freedom; AIC=Akaike Information Criterion

^1^ p-value for a hypothesis test of no difference between gender/education

^2^ Lower AIC indicates better model fit. Change in AIC compared to model with no interaction (Table S2); a change of 5 or less generally considered to indicate no improvement in model fit.

^3^ or equivalent qualifications.
